# Combined identification of lncRNA NONHSAG004550 and NONHSAT125420 as a potential diagnostic biomarker of perinatal depression

**DOI:** 10.1002/jcla.23890

**Published:** 2021-07-15

**Authors:** Li Wang, Ming Zhang, Haiyan Zhu, Liying Sun, Bin Yu, Xuelian Cui

**Affiliations:** ^1^ Department of Healthcare Changzhou Maternity and Child Health Care Hospital Affiliated with Nanjing Medical University Changzhou China; ^2^ Department of Clinical Laboratory Changzhou Maternity and Child Health Care Hospital Affiliated with Nanjing Medical University Changzhou China; ^3^ Department of Genetic Laboratory Changzhou Maternity and Child Health Care Hospital Affiliated with Nanjing Medical University Changzhou China

**Keywords:** diagnostic biomarker, long noncoding RNA (lncRNA), NONHSAG004550, NONHSAT125420, perinatal depression (PD)

## Abstract

**Background:**

Perinatal depression (PD) is one of the most common complications of pregnancy, and timely diagnosis and treatment are still challenging in China due to the scarcity of psychiatrists. This study aimed to investigate whether long noncoding RNAs (lncRNAs) are potential diagnostic biomarkers of PD.

**Methods:**

Using RT‐PCR, six downregulated major depressive disorder (MDD)‐associated lncRNAs (NONSUSG010267, NONHSAT140386, NONHSAG004550, NONHSAT125420, NONHSAG013606, and NONMMUG014361) were assessed in 39 pregnant women with PD (PD group), 20 PD patients undergoing mindfulness‐integrated cognitive behavior therapy (MiCBT) (treatment group (TG)), and 51 normal pregnant women (normal control (NC) group) to identify significantly differentially expressed lncRNAs during the second trimester and at 42 days postpartum.

**Results:**

Compared with the NC group, the six lncRNAs were significantly downregulated in the PD group during the second trimester and at 42 days postpartum (*p*<0.01~0.001). Expression of NONHSAG004550 and NONHSAT125420 was significantly upregulated after MiCBT therapy in TG (*p*<0.01~0.001), and no significant differences were observed between TG and the NC group at 42 days postpartum (*p*>0.05). NONHSAG004550 and NONHSAT125420 were significantly differentially expressed in the PD group, and this expression was altered according to the amelioration of depressive symptoms. Receiver operating characteristic (ROC) curve analysis revealed that the two lncRNAs combined had a good value in predicting PD, with an area under the curve (AUC) of 0.764 (95% confidence interval (CI): 0.639–0.888).

**Conclusion:**

The combination of lncRNAs NONHSAG004550 and NONHSAT125420 is a novel potential diagnostic biomarker of PD.

## Graphical abstract

Background: Perinatal depression (PD) is one of the most common complications of pregnancy, and timely diagnosis and treatment are still challenging in China due to the scarcity of psychiatrists. This study aimed to investigate whether long noncoding RNAs (lncRNAs) are potential diagnostic biomarkers of PD. Methods: Using RT‐PCR, six downregulated major depressive disorder (MDD)‐associated lncRNAs (NONSUSG010267, NONHSAT140386, NONHSAG004550, NONHSAT125420, NONHSAG013606, and NONMMUG014361) were assessed in 39 pregnant women with PD (PD group), 20 PD patients undergoing mindfulness‐integrated cognitive behavior therapy (MiCBT) (treatment group (TG)), and 51 normal pregnant women (normal control (NC) group) to identify significantly differentially expressed lncRNAs during the second trimester and at 42 days postpartum. Results: Compared with the NC group, the six lncRNAs were significantly downregulated in the PD group during the second trimester and at 42 days postpartum (P<0.01~0.001). Expression of NONHSAG004550 and NONHSAT125420 was significantly upregulated after MiCBT therapy in TG (P<0.01~0.001), and no significant differences were observed between TG and the NC group at 42 days postpartum (P>0.05). NONHSAG004550 and NONHSAT125420 were significantly differentially expressed in the PD group, and this expression was altered according to the amelioration of depressive symptoms. Receiver operating characteristic (ROC) curve analysis revealed that the two lncRNAs combined had good value in predicting PD, with an area under the curve (AUC) of 0.764 (95% confidence interval (CI): 0.639‐0.888). Conclusion: The combination of lncRNAs NONHSAG004550 and NONHSAT125420 is a novel potential diagnostic biomarker of PD.

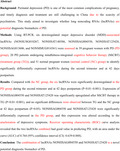

## 1 INTRODUCTION

Perinatal depression (PD), one of the most common complications of pregnancy, is defined as major depression with peripartum onset occurring during pregnancy or within four weeks of delivery.^1^ More than half of the women with PD have depressive symptoms before or during pregnancy,^2^ and up to 13% of women experience symptoms of depression during pregnancy or postpartum.^3^ PD is associated with behavioral problems, learning difficulties, and psychiatric illness in children.^4^, ^5^, ^6^, ^7^ Human studies have suggested that the second trimester is the most sensitive period, when exposure to stressful life events increases the risk of illness in offspring.^8^, ^9^

In China, pregnant women are reluctant to go to psychiatric hospitals. As a result, timely PD diagnosis in maternal and child health hospitals is insufficient. Maternal depression during pregnancy may be associated with cord blood DNA methylation,^10^, ^11^ and antidepressant medication^10^ or psychotherapy^12^, ^13^ during pregnancy may have effects on the epigenome of the child. Thus, it is important to investigate potential laboratory diagnostic biomarkers to detect PD early in maternal and child health hospitals, specifically in the second trimester rather than in the postpartum period.

Epigenetics is the study of potentially heritable molecular modifications to DNA and histone proteins that can affect gene expression without changing the underlying DNA sequence.^9^ Epigenetic processes during fetal development are one pathway through which environmental factors may affect phenotypes later in life. As a type of epigenetic regulatory molecule, long noncoding RNAs (lncRNAs) play important roles in disease pathologic processes, and the use of lncRNAs as novel biomarkers in various cancer types and nervous system diseases has been investigated through clinical research programs.^14^, ^15^, ^16^, ^17^ lncRNAs may also contribute to the pathophysiology of major depression because of their association with cognitive disorders and synaptic plasticity.

Our previous studies showed that six downregulated lncRNAs (NONSUSG010267, NONHSAT140386, NONHSAG004550, NONHSAT125420, NONHSAG013606, and NONMMUG014361) have diagnostic and differential diagnostic value and participated in many central nervous system functions in major depressive disorder (MDD),^18^, ^19^ as illustrated in the Supplementary File. By comparing the expression of these six lncRNAs between PD patients, PD patients who voluntarily accepted mindfulness‐integrated cognitive behavior therapy (MiCBT),^20^ and normal pregnant women, the present study was primarily designed to explore whether certain differentially expressed lncRNAs could be potential diagnostic biomarkers of PD.

## 2 MATERIALS AND METHODS

### 2.1 Participants

A total of 63 drug‐free spontaneously pregnant women in the second trimester (14 weeks to 27^+^ ^6^ weeks pregnant) who met the criteria in the Diagnostic and Statistical Manual, 5^th^ Edition, (DSM‐V) for PD disorder were enrolled from Changzhou Maternity and Child Healthcare Hospital from January 2017 to September 2019. Patients with cognitive impairment, in vitro fertilization and embryo transfer (IVF‐ET), physical illness (gestational diabetes, gestational hypertension, hyperthyroidism, hypothyroidism, and anemia Hb<100 g/L), or serious suicidal ideation or attempts were excluded. The 63 pregnant women were all given mental health education about PD; 43 of the women who were reluctant to receive treatment were placed into the PD group, and 20 women who voluntarily accepted MiCBT were placed in the treatment group (TG).

Fifty‐five spontaneously pregnant women in the second trimester who did not meet the criteria for PD according to the DSM‐V were considered normal controls (NCs). Pregnant women with cognitive impairment; IVF‐ET; or physical illness, such as gestational diabetes, gestational hypertension, hyperthyroidism, hypothyroidism, and anemia Hb<100 g/L, were excluded.

Blood was not collected from 4 PD patients and 4 NC patients at 42 days postpartum. Therefore, their data were excluded from the PD and NC groups in both the second trimester and 42 days postpartum.

Information about age, ethnicity, and education was collected from all subjects. All subjects included in the study were nonsmokers and nondrinkers to avoid these possible influences.

### 2.2 EPDS assessment

All the subjects were assessed according to the Edinburgh Postnatal Depression Scale (EPDS) in the second trimester and at 42 days postpartum. The EPDS has been used to evaluate maternal depressive symptoms during and after pregnancy.^20^ The maximum score is 30, a score of 0–10 is considered normal, 11–12 suggests possible depression, and 13 or higher indicates depression.^21^

### 2.3 MICBT therapy

MiCBT was used to intervene in the depressive symptoms of pregnant women.^20^, ^22^ The MiCBT program in this study was implemented via eight 90‐minute sessions in the second trimester according to the voluntary principle. The content of each session was as follows:

Session 1. An overview of MiCBT, the flow of the program, and the contents of the next sessions.

Session 2. The basic principles of mindfulness, the components of CBT, and mindful breathing.

Session 3. Mindful breathing (continued), step‐by‐step body scanning exercises, and awareness of visceral sensations.

Session 4. Body scanning exercises (continued), behavior therapy techniques (such as problem‐solving), and the relationship between mindfulness and CBT.

Session 5. Body scanning exercises (continued).

Session 6. Interpersonal skills, assertiveness, and role play.

Session 7. Acceptance and management of suffering in daily life.

Session 8. Review and evaluation.^23^

The intervention was performed by an MSc in clinical psychiatry (corresponding author) who had received specialized training in this area under the supervision of a PhD in clinical psychology. Intervention sessions were held at Changzhou Maternity and Child Healthcare Hospital.

### 2.4 Collection of human blood and RNA samples

Whole blood samples from all subjects were collected using EDTA anticoagulant tubes and centrifuged (400×g for 40 min) within 2 hours. Peripheral blood mononuclear cell (PBMC) lymphocytes and monocytes were isolated from the blood using density gradient centrifugation and then stored at −80℃ until use. RNA was extracted from PBMCs using TRIzol (Invitrogen, Carlsbad, CA, USA) and purified using a QIAGEN RNeasy kit (Qiagen, Valencia, CA, USA) according to the manufacturer's instructions. RNA extraction and storage were carried out as previously described.^19^

### 2.5 RT‐PCR analysis of lncRNAs

Quantitative RT‐PCR was used to test six downregulated MDD lncRNAs (NONSUSG010267, NONHSAT140386, NONHSAG004550, NONHSAT125420, NONHSAG013606, and NONMMUG014361). cDNA was synthesized using a TaqMan RNA Reverse Transcription Kit (ABI., USA) according to the manufacturer's protocol. Each RT reaction included 10 μl of total RNA, 4.16 μl of nuclease‐free water, 3.0 μl of TaqMan MicroRNA assay, 1 μl multiscribe reverse transcriptase, 0.19 μl RNase inhibitor, 1.5 μl 10×RT buffer, and 0.15 μl dNTPs in a total volume of 15 μl. Reactions were carried out under the following conditions: 30 mins at 16℃, 30 mins at 42℃, 5 mins at 85℃, and 10 mins at 4℃. Each sample was tested in duplicate. Real‐time PCR was performed using an Applied Biosystems 7900HT Real‐Time PCR System (Applied Biosystems, Inc., USA) and was implemented according to previously described methods.^19^

DataAssist v3.0 software and SDS 2.3 software (Applied Biosystems, Inc.) were used to collect the data, and the expression levels of lncRNAs were calculated after normalization to β‐actin expression. The primers used in the study are listed in Supplementary File Table 1.

**TABLE 1:** Demographics of perinatal depression patients and healthy controls.

	PD	TG	NC	Comparison	
				F	P value
Number	39	20	51		
Age (year)	30.67(5.8)	31.66(4.9)	31.69 (6.1)	0.80	0.465
Education (year)	15.7 (3.0)	15.2(2.6)	14.3 (2.4)	1.20	0.236
EPDS score (second trimester)	18.5(3.9)	19.1 (3.7)	6.2(1.5)	7.73	<0.01
EPDS score(42 days postpartum)	19.3 (4.0)	7.6 (2.7)	6.1 (1.2)	3.85	<0.05
Ethnic	Han	Han	Han		

Abbreviations: EPDS, Edinburgh postnatal depression scale; NC, Normal control group; PD, Perinatal depression group; TG, Therapeutic group.

### 2.6 Statistical analysis

All statistical analyses were conducted using the SPSS 25.0 software package (Chicago, IL, USA) and GraphPad Prism 5.0 (GraphPad Software Inc., San Diego, CA, USA). Independent sample t‐tests, ANOVA, and post hoc (Tukey) tests were used to compare the demographic variables and lncRNA expression differences among the three groups both in the second trimester and at 42 days postpartum. The specificity and sensitivity of lncRNAs for detection of PD were assessed with receiver operating characteristic (ROC) curves. *p*<0.05 (two‐sided) was considered statistically significant.

## 3 RESULTS

### 3.1 Demographic data for patients in the PD group, TG, and NC group

According to ANOVA, no significant differences in age or education (*p*>0.05) were found among the three groups, though EPDS scores differed significantly (*p*<0.01). Indeed, there was a significant difference in the EPDS scores between the PD and NC groups and between TG and the NC group (t=2.56~2.78, *p*<0.01) in the second trimester but no significant difference between TG and the NC group (t=1.02, *p*>0.05) at 42 days postpartum. The demographic data for the subjects are shown in Table 1.

### 3.2 Comparison of expression of six lncRNAs between the groups in the second trimester

As noted in Table 2 (in the Supplementary File), according to a Tukey analysis, expression of the six tested lncRNAs (NONSUSG010267, NONHSAT140386, NONHSAG004550, NONHSAT125420, NONHSAG013606, and NONMMUG014361) in the three groups was significantly different in the second trimester: the F value ranged from 8.448 to 15.524, and the P value was 0.000.

**FIGURE 1:**
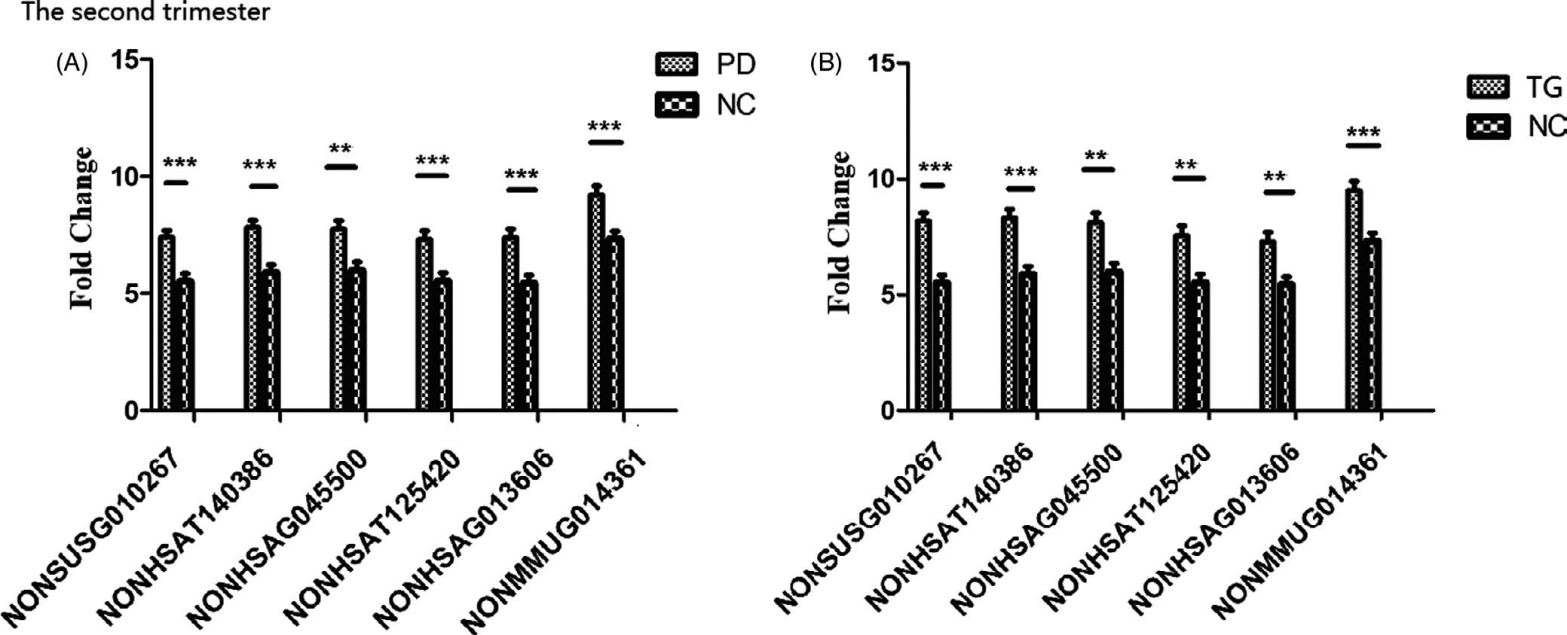
Comparison of six lncRNA expression levels among the three groups in the second trimester. The expression of the six lncRNAs was significantly downregulated in the PD group (A) and TG (B) in the second trimester compared with levels in the NC group. **p<0.01, ***p<0.001 compared to the NC

As indicated in Figure 1, according to a Tukey honest significant difference (HSD) test, the six lncRNAs were all significantly downregulated in the PD group and TG compared with the NC group (*p*=0.001~0.01); no significant differences were observed between the PD group and TG (*p*=0.375~0.988).

### 3.3 Comparison of expression of six lncRNAs between the groups at 42 days postpartum

**FIGURE 2:**
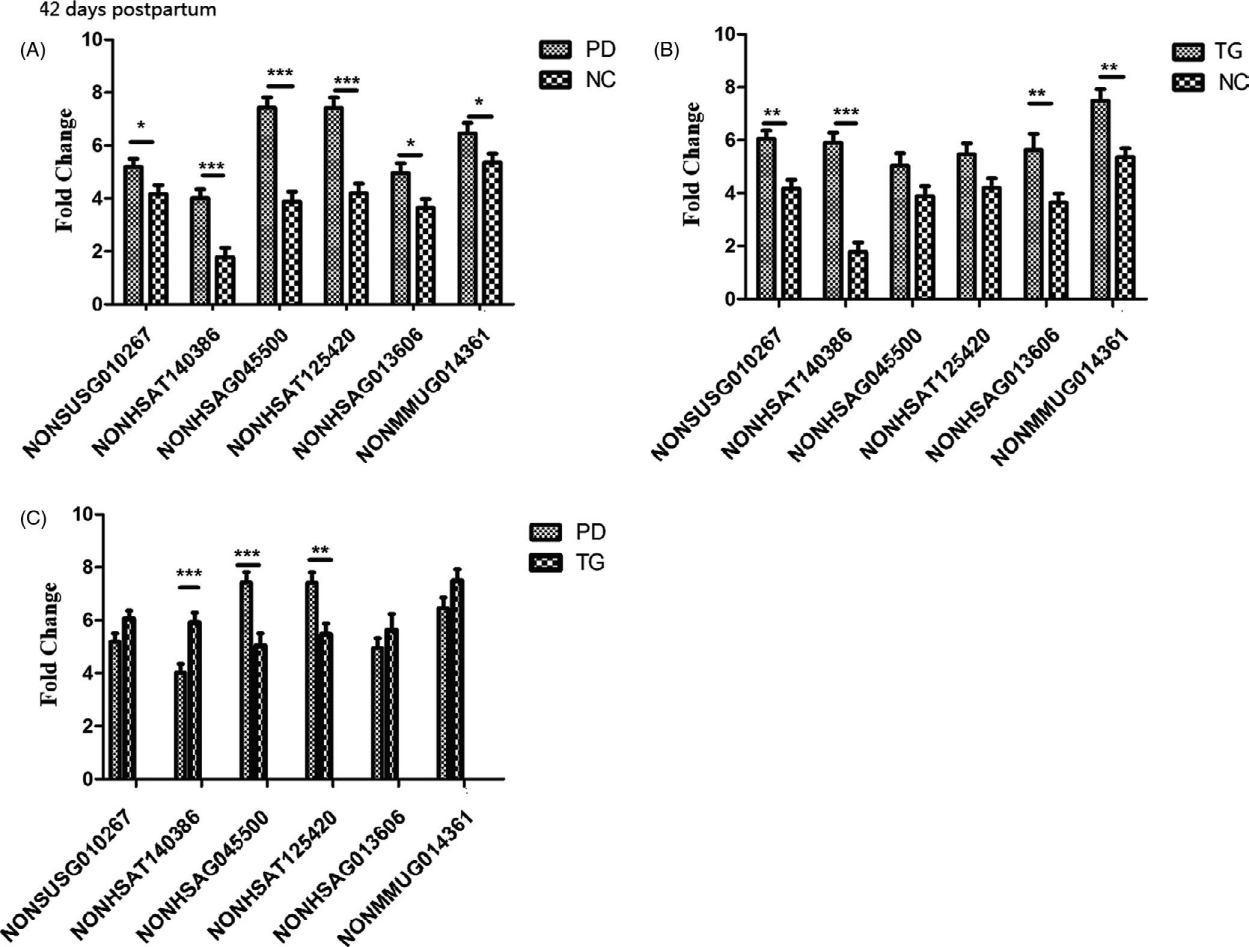
The expression of six lncRNAs in the three groups at 42 days postpartum. All six lncRNAs in the PD group (A) and four lncRNAs (NONSUSG010267, NONHSAT140386, NONHSAG013606, and NONMMUG014361) in the TG (B) were significantly downregulated compared with levels in the NC group. The expression of two lncRNAs, NONHSAG004550 and NONHSAT125420, was upregulated in the TG (C), and no significant differences were observed between the TG and NC group at 42 days postpartum (B). *p<0.05, **p<0.01, ***p<0.001

As demonstrated in Table 3 (in the Supplementary File), according to a Tukey analysis, expression of six lncRNAs was significantly different among the three groups (F=5.947–27.232, *p*=0.000–0.004) at 42 days postpartum. Through a Tukey HSD test, compared with the NC group, the six lncRNAs were significantly downregulated in the PD group (*p*=0.000–0.035) and four of the lncRNAs (NONSUSG010267, NONHSAT140386, NONHSAG013606, and NONMMUG014361) were significantly downregulated in TG (*p*=0.000–0.008). Additionally, expression of two of lncRNAs, NONHSAG004550 (*p*=0.193) and NONHSAT125420 (*p*=0.119), was upregulated in TG, and no significant differences were found between TG and the NC group at 42 days postpartum (*p*>0.05), as illustrated in Figure 2.

### 3.4 Effect of hormones on expression of six lncRNAs

After delivery, expression of the six lncRNAs in the NC group (t=2.83~8.37, *p*<0.01~0.001) and four (NONSUSG010267, NONHSAT140386, NONHSAG013606, and NONMMUG014361) in the PD group (t=4.609~8.613, *p*<0.001) was upregulated and significantly different from the levels in the second trimester. In the PD group, expression of NONHSAG004550 (t=0.180, *p*>0.05) and NONHSAT125420 (t=0.5858, *p*>0.05) was upregulated to some extent but not significantly different compared with the levels in the second trimester.

### 3.5 Predictive value of NONHSAG004550 and NONHSAT125420 combined for perinatal depression

**FIGURE 3:**
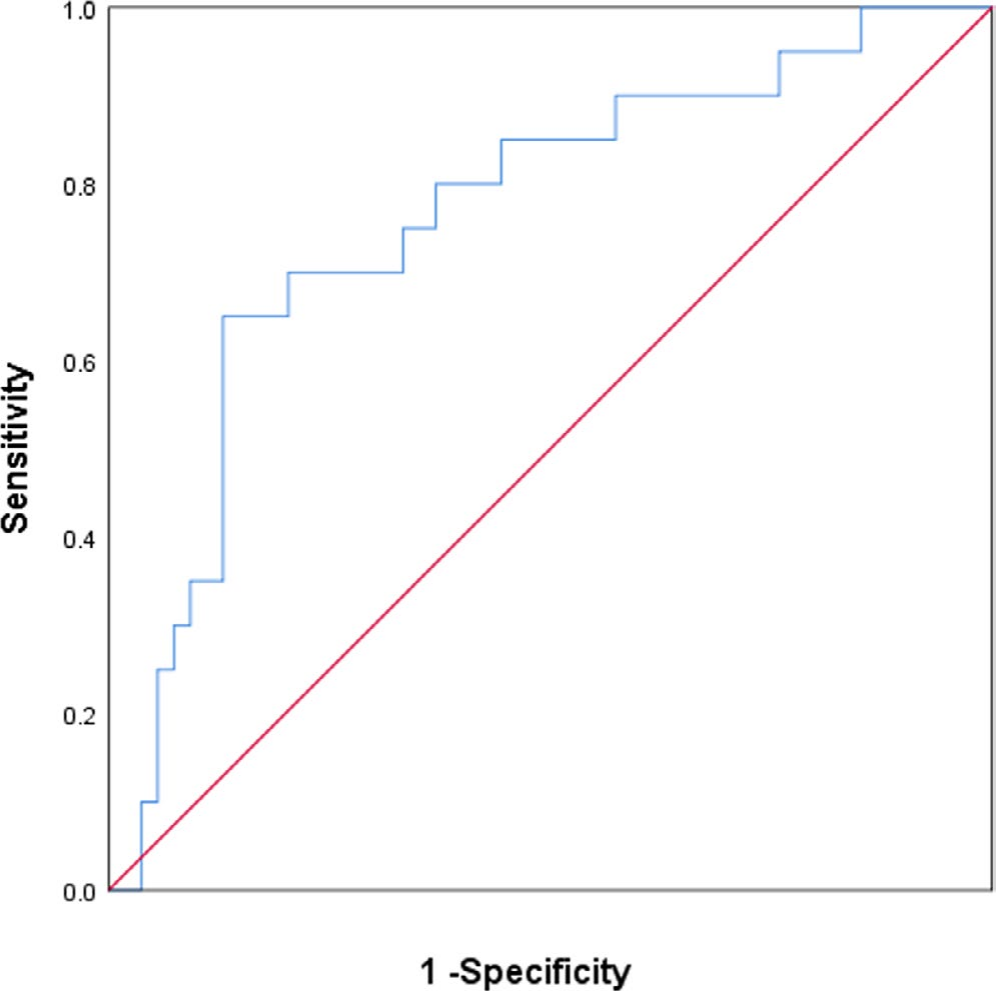
The combined receiver operating characteristic curve (ROC) of the two lncRNAs

ROC curve analysis revealed that the combination of lncRNA NONHSAG004550 and NONHSAT125420 had good value in predicting PD, with an area under the curve (AUC) of 0.764 (95% confidence interval (CI): 0.639–0.888, Figure 3), a sensitivity of 0.650 and a specificity of 0.870.

## 4 DISCUSSION

According to a previous systematic review, the prevalence of major depression is estimated to be as high as 11% during pregnancy and 13% in the first 3 months postpartum (period prevalence rates are 18.4% and 19.2%, respectively) in high‐income countries (HICs)^24^ and is higher in low‐ and middle‐income countries.^25^ Age and education are not risk factors for PD development.^26^ In this study, we found no significant differences in age or education among the three groups, which was consistent with previous research.

The second trimester (13 w‐27 w) is a sensitive period in which the mother's mental illness can affect the development of fetal organ function.^27^ Psychiatrists are scarce in China, especially in women and child health hospitals. Therefore, PD in women is often not detected in the early stages. We aimed to explore a laboratory indicator that could potentially serve as a diagnostic biomarker to screen for PD as early as the second trimester. The indicator should have three traits. First, the indicator should be significantly different in women with PD than in normal women. Second, the indicator should return to the normal range after relief of depressive symptoms. Third, the indicator must be specific and not affected by hormones. In this study, we first compared expression of six lncRNAs in the second trimester among three groups and found that all six were all significantly downregulated in the PD group and TG compared with the NC group, with no significant differences between the PD group and TG, as demonstrated in Figure 1 and Table 2 (in the Supplementary File).

The effects of psychotherapy on mental disorders have been revealed on multiple levels, including epigenetic, neurocircuitry, and neuroendocrine impacts.^12^, ^13^ Psychotherapy can change DNA methylation in the periphery and influence epigenetic processes.^28^ In the present study, MiCBT, a safe and nonpharmaceutical intervention, was implemented in TG in the second trimester. After childbirth, the EPDS scores in TG were not significantly different from those in the NC group at 42 days postpartum, but there was a significant difference between the PD and NC groups, as indicated in Table 1, which suggests that MiCBT was effective in this study. Subsequently, we compared the expression of the six lncRNAs between the three groups again at 42 days postpartum, and the results were different from those in the second trimester, as demonstrated in Table 3 and Figure 2. The lncRNAs NONHSAG004550 and NONHSAT125420 were upregulated in the TG but showed no significant difference between TG and the NC group at 42 days postpartum. However, the other four lncRNAs were still significantly downregulated in both the PD group and TG compared with the NC group, which preliminarily confirmed that the onset and relief of depressive symptoms might not be the main cause of changes in the expression of the four lncRNAs.

Hormones play an important role in epigenetics. Unmethylated genomic regions that encode feedback regulatory modules and differentially recruit RNA polymerase II and acetylases/deacetylases are involved in the epigenetic mechanism underlying the crosstalk between steroid hormones and growth factors.^29^ Epigenetic remodeling of chromatin structure and the interplay with noncoding RNA have emerged as novel thyroid hormone‐dependent pathways in the regulation of cardiovascular physiology.^30^ DNA methylation variation at HP1BP3 and TTC9B was found to be modified by estrogen exposure in the rodent hippocampus,^31^ and postpartum depression was shown to be mediated by differential gene expression and epigenetic sensitivity to pregnancy hormones.^32^ In the present study, we assessed the effects of hormonal changes from pregnancy to the postpartum period, such as a fall or rise in the levels of estrogens and progesterone, a raised prolactin level, a raised cortisol level, and thyroxin and triiodothyronine levels, on the expression of six lncRNAs. The results showed four lncRNAs (NONSUSG010267, NONHSAT140386, NONHSAG013606, and NONMMUG014361) to be significantly upregulated in the PD and NC groups after childbirth, which indicates that hormone variation might cause changes in the expression of these four lncRNAs. In contrast, expression of NONHSAG004550 and NONHSAT125420 might primarily be regulated by depressive symptoms and psychotherapy but not hormone changes.

NONHSAT125420 and NONHSAG004550 expression profiles have been evaluated in brain, ovary, breast, and adipose tissues. Through Kyoto Encyclopedia of Genes and Genomes (KEGG) pathway and Gene Ontology (GO) analysis, the functions of the two lncRNAs were found to involve protein transport, protein complex biogenesis, and protein complex assembly, and they might participate in the development of Alzheimer's disease, Parkinson's disease, and long‐term depression. Abnormal reuptake of the neurotransmitters 5‐HT and norepinephrine is well known to be involved in the pathogenesis of depression. Thus, we hypothesized that the lncRNAs NONHSAG004550 and NONHSAT125420 might be involved in neural pathways related to 5‐HT or norepinephrine metabolism. Whether NONHSAG004550 and NONHSAT125420 can regulate the level of 5‐HT in the synaptic cleft and SERT expression needs further investigation.

In summary, the lncRNAs NONHSAG004550 and NONHSAT125420 seem to have the three traits of potential diagnostic biomarkers of PD. In the clinic, NONHSAG004550 and NONHSAT125420 expression levels might be tested in pregnant women in the second trimester to help doctors predict the possibility of PD and make timely referrals for therapy.

Some limitations of the study must be acknowledged. First, this was an open‐label study based on voluntary reports from patients. Hence, further verification is needed in future randomized double‐blind studies. Second, the results of this study might be biased due to the limited sample size, and the sample size should be expanded to obtain the reference range of the biomarkers. Third, the expression of the six lncRNAs was not tested before the women became pregnant. Thus, we could not determine the prepregnancy variation in lncRNAs, making the analysis of the effect of hormone variation on lncRNA expression incomplete.

## CONFLICTS OF INTEREST

The authors declare no conflicts of interest.

## DATA AVAILABILITY STATEMENT

The data used to support the findings of the present study are available from the corresponding author upon request.

## Supporting information

Supplementary Material

Supplementary Material
