# Supplementary material for: Combined identification of lncRNA NONHSAG004550 and NONHSAT125420 as a potential diagnostic biomarker of perinatal depression
Source: J Clin Lab Anal. 2021 Jul 15;35(8):e23890. doi: 10.1002/jcla.23890 (PMC8373316; doi:10.1002/jcla.23890)
Supplement: Supplementary file 1 — Supplementary Material [file JCLA-35-e23890-s002.doc]

**Supplementary File**

**The corresponding probe's names as demonstrated in our previous studies are as follows:**

**ENST00000517573→****NONHSAT125420；CONS_00019174→****NONSUSG010267；**

**ENST00000566208→****NONHSAT140386；NONHSAT034045→****NONHSAG013606**

**NONHSAT142707→** **NONMMUG014361；NONHSAG004550→NONHSAG004550**

**TABLE 1 Primers used for RT-PCR**.

| **Gene** | **Forward （5’-3’）** | **Reverse （5’-3’）** |
| --- | --- | --- |
| NONSUSG010267 | GGGGTACCGTTGCCAAATGGGTTTTG | CGGGATCCTTTGGAGAAAATATTTTATTTAC |
| NONHSAT140386 | ATGGCAGGGCCTTAGTC | GGTCAGGCTGTGGTCT |
| NONHSAG004550 | ACCTGTTACCCTGGAAGT | CACATTAAGGCTGTGAGC |
| NONHSAT125420 | CTCTTGGTGCTGGCTGGT | GCCAGTAGATGCGAGTTG |
| NONHSAG013606 | CTCGCTTCGGCAGCAC | AACGCTTCACGAATTGCGT |
| NONMMUG014361 | ATCTGCAAGCCAGGAAGAGTC | CTTGCTTGATGCTTTGGTCTGT |

**TABLE 2** Comparison of six lncRNAs expression between three groups in the second trimester (x±s)

|  | NC | PD | | TG | | Comparison | |  |
| --- | --- | --- | --- | --- | --- | --- | --- | --- |
|  |  |  |  |  |  | F | P-value | |
| NONSUSG010267 | 5.5167±2.4976 | 7.4089±1.8509 | 8.1870±1.5828 | | 15.524 | | 0.000 |  |
| NONHSAT140386 | 5.8935±2.5745 | 7.8252±1.9632 | 8.3338±1.6625 | | 13.281 | | 0.000 |  |
| NONHSAG004550 | 5.9857±2.7349 | 7.7466±2.4336 | 8.1237±1.8835 | | 8.448 | | 0.000 |  |
| NONHSAT125420 | 5.4780±2.6318 | 7.3238±2.3780 | 7.5450±1.9107 | | 9.160 | | 0.000 |  |
| NONHSAG013606 | 5.4420±2.5105 | 7.3861±2.4359 | 7.2902±1.8815 | | 9.425 | | 0.000 |  |
| NONMMUG014361 | 7.3255±2.4992 | 9.2161±2.4939 | 9.5096±1.7611 | | 10.211 | | 0.000 |  |

**TABLE 3** Comparison of six lncRNAs expression between three groups at 42 days postpartum (x±s)

|  | NC | PD | | TG | | Comparison | |  |
| --- | --- | --- | --- | --- | --- | --- | --- | --- |
|  |  |  |  |  |  | F | P-value | |
| NONSUSG010267 | 4.1667±2.4976 | 5.1857±1.9599 | 6.0453±1.3993 | | 6.259 | | 0.003 |  |
| NONHSAT140386 | 1.7835±2.5745 | 4.0156±2.0403 | 5.9005±1.7447 | | 27.232 | | 0.000 |  |
| NONHSAG004550 | 3.8857±2.7349 | 7.4295±2.4629 | 5.0417±2.0586 | | 22.373 | | 0.000 |  |
| NONHSAT125420 | 4.2004±2.7067 | 7.4194±2.4396 | 5.5012±1.9731 | | 18.864 | | 0.000 |  |
| NONHSAT034045 | 3.6420±2.510 | 4.9549±2.3287 | 5.6314±2.7331 | | 5.947 | | 0.004 |  |
| NONHSAT142707 | 5.3618±2.5155 | 6.4633±2.4820 | 7.4894±1.9659 | | 6.301 | | 0.003 |  |

**TCONS_00019174**


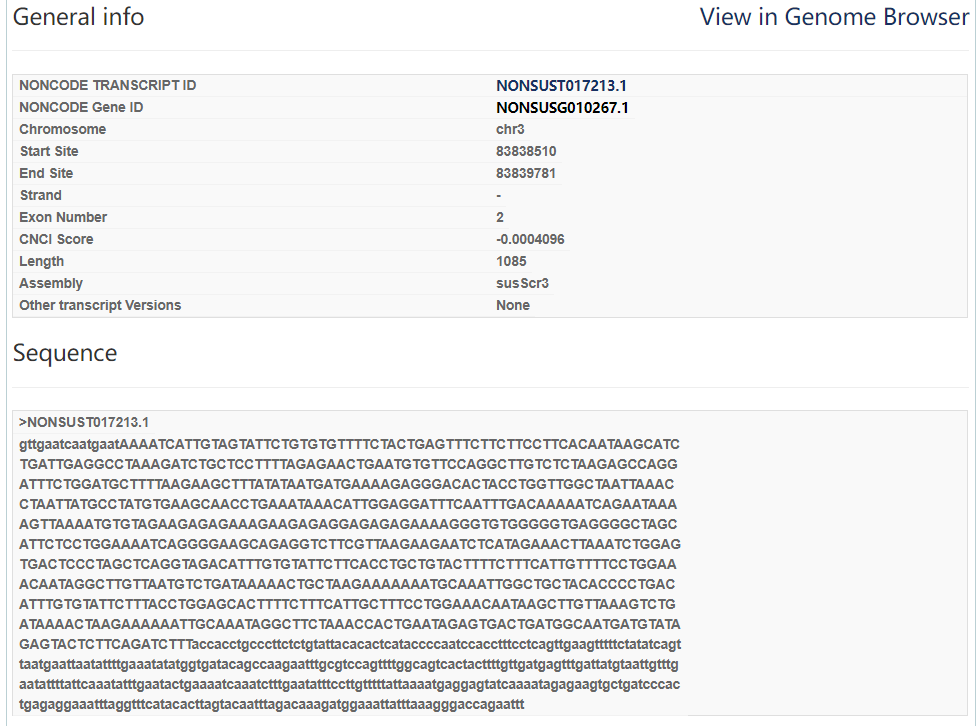


ENST00000566208


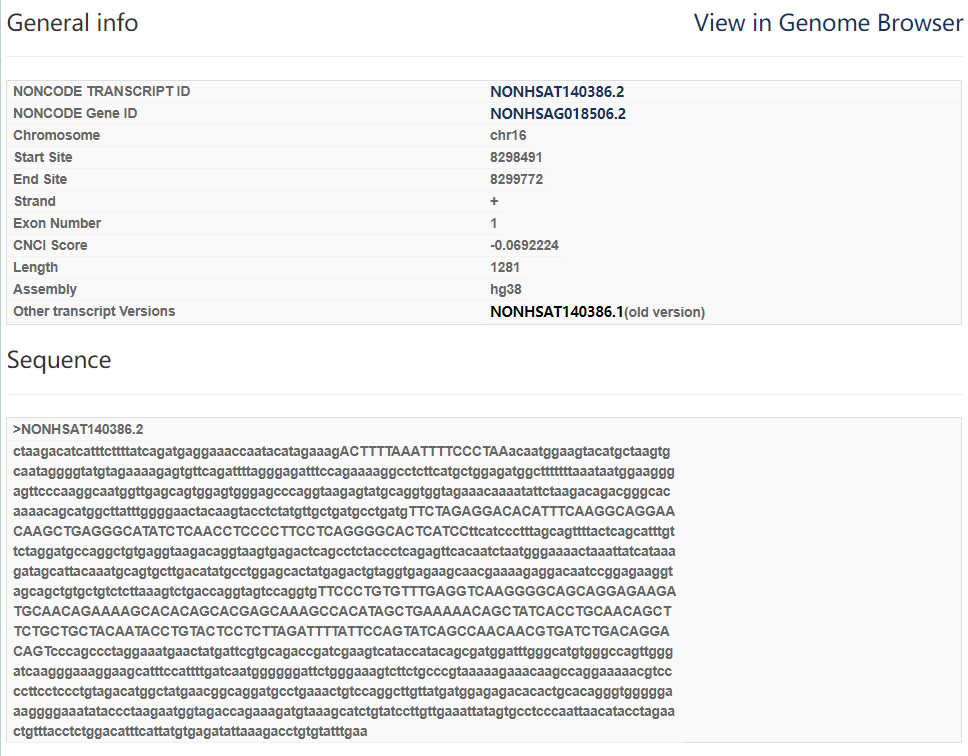


NONHSAG004550


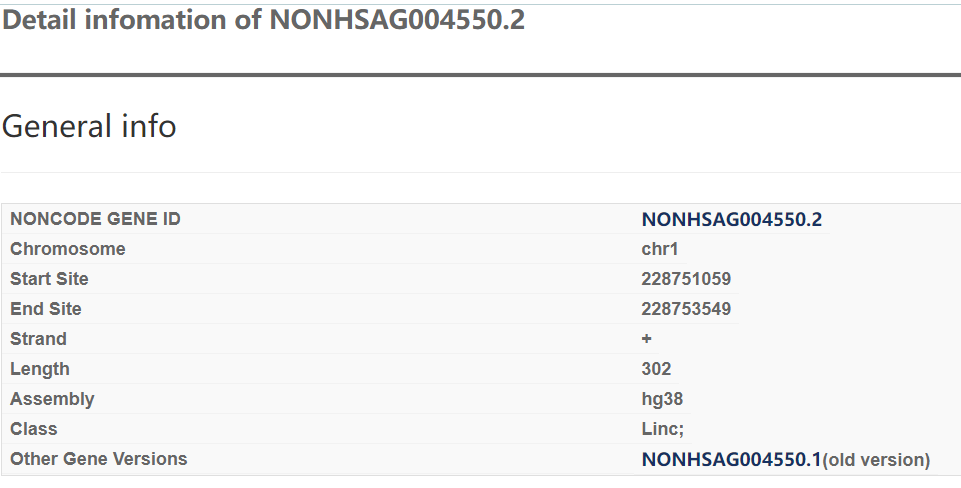


TTTTATTTAAACCCGAGAGAGAGGAGGGCCCTGCTTCCTCCACCACCCCACTGTGGGTGTGGTGGGAACTGTATCTCCTACACCACAGGTGCCCAATGGTCTGTGGAATGATTGCTAGCCAAGGCCATCTTTTAAATGATTCCCCACCATGAACCATGGAGGCTGAACTACATCAATGGGTCTCTCACCTGAATGTATGTGTGGGTCGCCTGGGGTTTCGCTAAGATGCAGATGCTGATTCAGAAACTCCCGGTTGAGACCGAGGCTCTGCATTGCTGACCAGGGCCCAGGTGACTCCAGGATTGCTGTCCAGGTAATGCCTTGGGACCAGAGCCAGGGGCCAGACATTCTGTCCTGTCCTCCAGACCCAGCATCCCACAGCAGGAGCACACAGCTGTGG

**ENST00000517573**


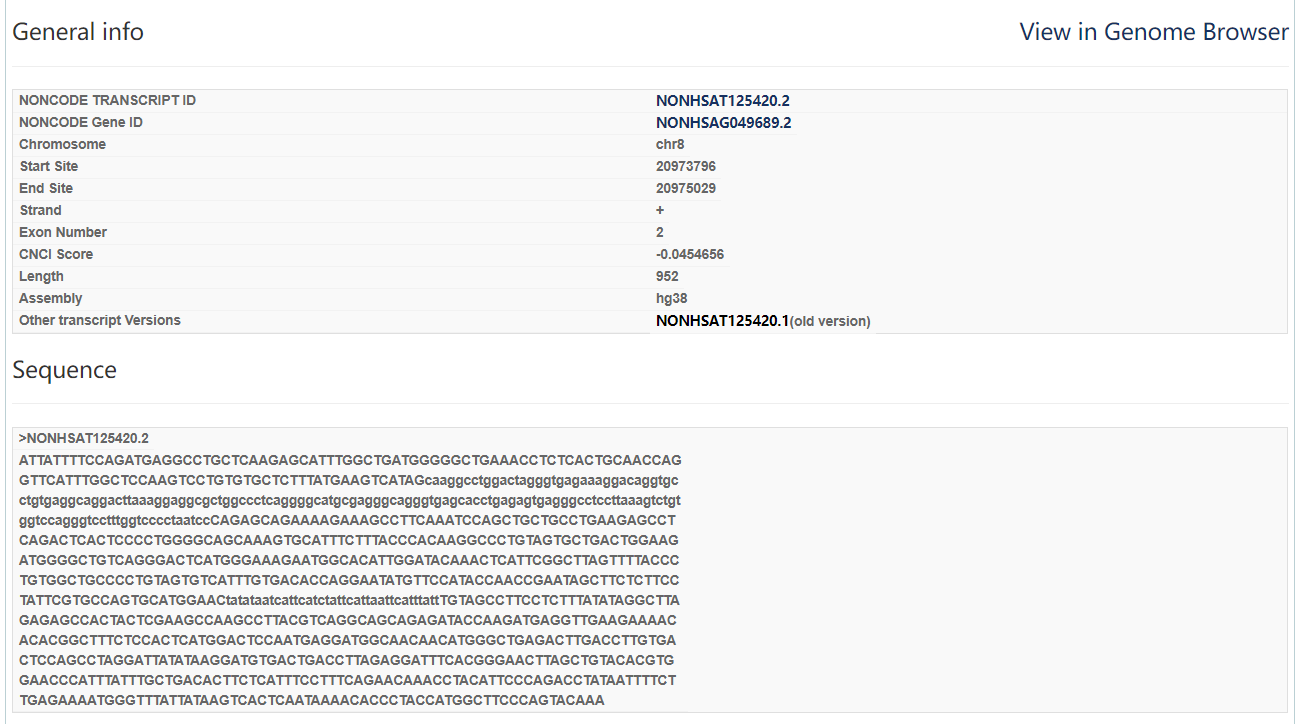


**NONHSAT034045**


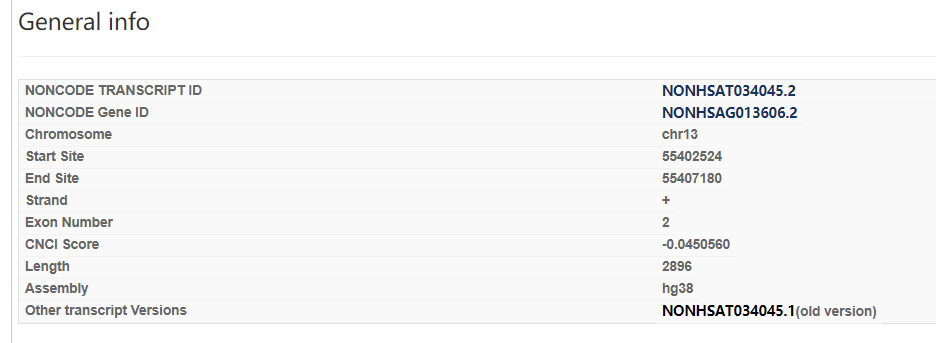


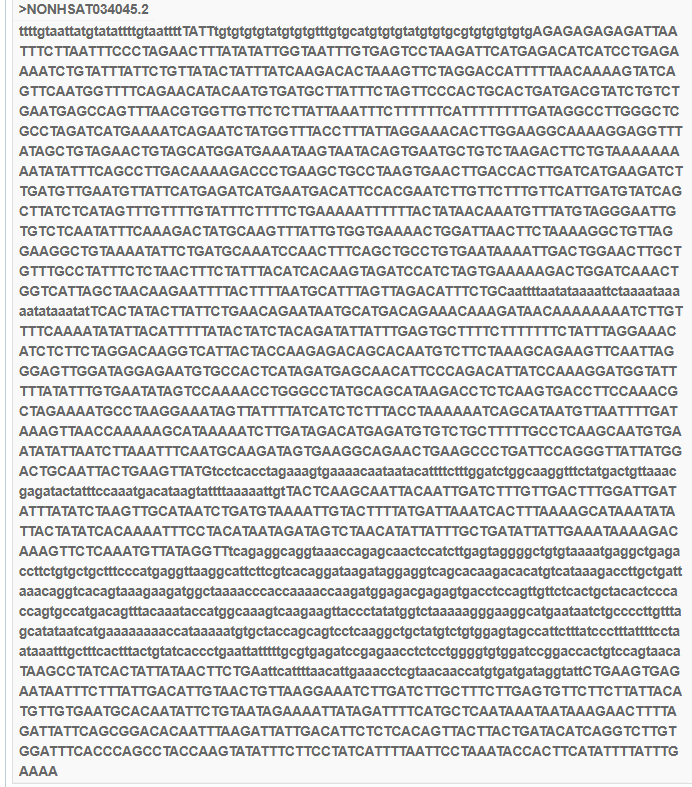


Total RNA isolation

- **Cell Lysis and Tissue Disruption**

**1. Collect 10^2^–10^7^cells or 0.5–250 mg tissue; wash cells in cold PBS**

**Suspension cells:** Count the cells, then pellet 10^2^–10^7^cells at low speed, and discard the culture medium. Wash the cells by resuspending in ~1 mL PBS, and repelleting. Place the washed cells on ice.

**Adherent cells:** do one of the following

• Aspirate and discard the culture medium, and rinse with PBS. Place the culture plate on ice.

• Trypsinize cells to detach them and count. Then inactivate the trypsin, pellet the cells, and discard the supernatant (following the method employed in your lab for the cell type). Wash the cells by gently resuspending in ~1 mL PBS, and pelleting at low speed. Place the cells on ice.

**Tissue samples:**For good yield of intact RNA, it is very important to obtain tissue quickly and to limit the time between obtaining tissue samples and inactivating RNases.

a. Obtain tissue and remove as much extraneous material as possible, for example remove adipose tissue from heart, and remove gall bladder from liver. The tissue can be perfused with cold PBS if desired to eliminate some of the red blood cells.

b. If necessary, quickly cut the tissue into pieces small enough for either storage or disruption. Weigh the tissue sample.

c. Freeze the sample in liquid nitrogen—tissue pieces must be small enough to freeze in a few seconds. When the liquid nitrogen stops churning, it indicates that the tissue is completely frozen. Once frozen, remove the tissue from the liquid nitrogen and store it in an airtight container below –70°C.

**2. Disrupt samples in 600 μL Lysis/Binding**

a. Place 10 volumes of Lysis/Binding Buffer per tissue mass into a plastic weigh boat or tube on ice.

b. Using a prechilled metal spatula, scrape the powdered tissue into the Lysis/Binding Buffer, and mix rapidly.

c. Transfer the mixture to a vessel for homogenization and process the mixture to homogeneity.

- **Organic Extraction**

**1. Add 1/10 volume of miRNA Homogenate Additive, incubate 10 min on ice**

a. Add 1/10 volume of miRNA Homogenate Additive to the cell or tissue lysate (or homogenate), and mix well by vortexing or inverting the tube several times. For example, if the lysate volume is 300 μL, add 30 μL miRNA Homogenate Additive.

b. Leave the mixture on ice for 10 min.

**2. Extract with a volume of Acid-Phenol: Chloroform equal to the initial lysate volume**

a. Add a volume of Acid-Phenol:Chloroform that is equal to the lysate volume before addition of the miRNA Homogenate Additive.

b. Vortex for 30–60 sec to mix.

c. Centrifuge for 5 min at maximum speed (10,000 x g) at room temperature to separate the aqueous and organic phases. After centrifugation, the interphase should be compact; if it is not, repeat the centrifugation.

**3. Recover the aqueous phase; transfer the aqueous phase to a fresh tube**

Carefully remove the aqueous (upper) phase without disturbing the lower phase, and transfer it to a fresh tube. Note the volume removed.

- **Final RNA Isolation**

**1. Add 1.25 volumes 100% ethanol, and mix thoroughly**

Add 1.25 volumes of room temperature 100% ethanol to the aqueous phase

**2. Pass the lysate/ethanol mixture through a Filter Cartridge**

a. For each sample, place a Filter Cartridge into one of the Collection Tubes supplied.

b. Pipet the lysate/ethanol mixture (from the previous step) onto the Filter Cartridge. Up to 700 μL can be applied to a Filter Cartridge at a time, for samples larger than this, apply the mixture in successive applications to the same filter.

c. Centrifuge for ~15 sec to pass the mixture through the filter. Centrifuge at RCF 10,000x g (typically 10,000 rpm). Spinning harder than this may damage the filters. Alternatively, vacuum pressure may be used to pass samples through the filter.

d. Discard the flow-through, and repeat until all of the lysate/ethanol mixture is through th e filter. Reuse the Co llection Tube for the washing steps.

**3. Wash the filter with 700 μL miRNA Wash Solution 1**

Apply 700 μL miRNA Wash Solution1 (working solution mixed with ethanol) to the Filter Cartridge and centrifuge for ~5–10 sec or use a vacuum to pull the solution through the filter. Discard the flow-through from the Collection Tube, and replace the Filter Cartridge into the same Collection Tube.

**4. Wash the filter twice with 500 μL Wash Solution 2/3**

a. Apply 500 μL Wash Solution 2/3 (working solution mixed with ethanol) and draw it through the Filter Cartridge as in the previous step.

b. Repeat with a second 500 μL aliquot of Wash Solution 2/3.

c. After discarding the flow-through from the last wash, replace the Filter Cartridge in the same Collection Tube and spin the assembly for 1 min to remove residual fluid from the filter.

**5. Elute RNA with 100 μL 95°C Elution Solution or Nuclease-free Water**

Transfer the Filter Cartridge into a fresh Collection Tube (provided with the kit). Apply 100 μL of pre-heated (95°C) Elution Solution or nuclease-free water to the center of the filter, and close the cap. Spin for ~20–30 sec at maximum speed to recover the RNA.Collect the eluate (which contains the RNA) and store it at –70° C.

**LncRNA Microarray Protocol**

# Step 1 Total RNA Clean-up and RNA QC

**Kits and Reagents:**

RNasey Mini Kit (Qiagen p/n 74104):

**Procedure:**

1. Mix the following in a microcentrifuge tube:

| Redissolved RNA | ≤85ul |
| --- | --- |
| 10×Reaction buffer | 10ul |
| Baseline-ZERO DNase | 5ul |
| RNase-free water | X ul |
| **Total volume** | **100ul** |

1. Incubate at 37°C for 30 minutes.
2. Add 350 μl Buffer RLT, and mix well.
3. Add 250 μl ethanol (96–100%) to the diluted RNA, and mix well by pipetting. Do not centrifuge. Proceed immediately to step 5.
4. Transfer the sample (700 μl) to an RNeasy Mini spin column placed in a 2 ml collection tube. Close the lid gently, and centrifuge for 15 s at ≥8000 x g (≥10,000 rpm). Discard the flow-through.
5. Add 500 μl Buffer RPE to the RNeasy spin column. Close the lid gently, and centrifuge for 15 s at≥8000 x g (≥10,000 rpm) to wash the spin column membrane. Discard the flow-through.
6. Add 500 μl Buffer RPE to the RNeasy spin column. Close the lid gently, and centrifuge for 2 min at ≥8000 x g (≥10,000 rpm) to wash the spin column membrane.
7. Place the RNeasy spin column in a new 2 ml collection tube, and discard the old collection tube with the flow-through. Close the lid gently, and centrifuge at full speed for 1 min.
8. Place the RNeasy spin column in a new 1.5 ml collection tube. Add appropriate RNase-free water (please see report “RNA-QC”) directly to the spin column membrane. Close the lid gently, and centrifuge for 1 min at ≥8000 x g (≥10,000 rpm) to elute the RNA.
9. RNA quantification and quality control (Passed-please see report “RNA-QC”).

# Step 2 Prepare labeling reaction

**Kit:**

Quick Amp Labeling Kit, One-Color (Agilent p/n 5190-2305)

**Procedure:**

1. Add 200ng of total RNA (2.5μL) to a 1.5-mL microcentrifuge tube.
2. Add 0.8 μL of Random Primer.
3. Add 2 μL of Spike Mix.
4. Denature the primer and the template by incubating the reaction at 65°C in a circulating water bath for 10 minutes.
5. Place the reactions on ice and incubate for 5 minutes.
6. Immediately prior to use, gently mix the components listed in the following table for the cDNA Master Mix by adding in the order indicated, and put on ice.

|  | Volume(ul) per reaction |
| --- | --- |
| 5×First Strand Buffer | 2 |
| 0.1M DTT | 1 |
| 10mM dNTP mix | 0.5 |
| AffinityScript Rnase Block Mix | 1.2 |
| **Total volume** | **4.7** |

1. Briefly spin each sample tube in a microcentrifuge to drive down the contents from the tube walls and the lid. Return the tubes to ice.
2. Add 4.7 μL of cDNA Master Mix to each sample tube and mix by pipetting up and down.
3. Incubate samples at 40°C in a circulating water bath for 2 hours.
4. Move samples to a 70°C circulating water bath and incubate for 15 minutes.
5. Move samples to ice. Incubate for 5 minutes.
6. Spin samples briefly in a microcentrifuge to drive down tube contents from the tube walls and lid.
7. Immediately prior to use, gently mix the components listed in the following table in the order indicated for the Transcription Master Mix by pipetting at room temperature.

|  | Volume(ul) per reaction |
| --- | --- |
| Nuclease-free water | 0.75 |
| 5X Transcription Buffer | 3.2 |
| 0.1 M DTT | 0.6 |
| NTP mix | 1 |
| T7 RNA Polymerase | 0.24 |
| Cyanine-3-CTP | 0.21 |
| **Total volume** | **6** |

1. Add 6μL of Transcription Master Mix to each sample tube. Gently mix by pipetting.
2. Incubate samples in a circulating water bath at 40°C for 2 hours.

# Step 3 Purify the labeled/amplified RNA and labeled cRNA QC

**Kit:**

RNeasy Mini Kit (Qiagen p/n 74104)

**Procedure:**

1. Add 84 μL of nuclease-free water to your cRNA sample, for a total volume of 100 μL.
2. Add 350 μL of Buffer RLT and mix well by pipetting.
3. Add 250 μL of ethanol (100% purity) and mix thoroughly by pipetting. Do not centrifuge.
4. Transfer the 700 μL of the cRNA sample to an RNeasy mini column in a 2 mL collection tube. Centrifuge the sample at 4°C for 30 seconds at 13,000 rpm. Discard the flow-through and collection tube.
5. Transfer the RNeasy column to a new collection tube and add 500 μL of buffer RPE (containing ethanol) to the column. Centrifuge the sample at 4°C for 30 seconds at 13,000 rpm. Discard the flow-through. Re-use the collection tube.
6. Add another 500 μL of buffer RPE to the column. Centrifuge the sample at 4°C for 60 seconds at 13,000 rpm. Discard the flow-through and the collection tube.
7. Elute the cleaned cRNA sample by transferring the RNeasy column to a new 1.5 mL collection tube. Add appropriate RNase-free water (Passed-please see report “Labeling Efficiency-QC”) directly onto the RNeasy filter membrane. Wait 60 seconds, then centrifuge at 4°C for 30 seconds at 13,000 rpm.
8. Maintain the cRNA sample-containing flow-through on ice. Discard the RNeasy column.
9. Take 1.5 μl of each sample to determine the yield and specific activity by using the NanoDrop ND-2000 (Passed-please see report “Labeling Efficiency-QC”).

- From the main menu, choose MicroArray Measurement. Go to the Sample Type pull-down menu and select DNA-50.
- Blank the instrument with 1.5 μL of 1x labeling solution.
- Use 1.5 μL of purified labeled genomic DNA for quantitation. Measure the absorbance at A260nm (DNA), A550nm (cyanine 3).
- The specific activity (pmol dyes per μg genomic DNA) of the labeled genomic DNA can be obtained by the following calculation:

(Concentration of Cy3)

Specific Activity =——————————————— = pmol Cy3 per μg cRNA

(Concentration of cRNA) * 1000

* If the yield is <1.65 μg and the specific activity is <9.0 pmol Cy3 per μg cRNA do not proceed to the hybridization step. Repeat cRNA preparation.

# Step 4 Fragmentation and Hybridization

**Kit and Instruments:**

Agilent Gene Expression Hybridization Kit (Agilent p/n 5188-5242):

10X Blocking Agent

25X Fragmentation Buffer

2x GEx Hybridization Buffer HI-RPM

Hybridization Chamber, stainless (Agilent p/n G2534A)

Hybridization Chamber gasket slides (Agilent p/n G2534-60003)

Hybridization oven (Agilent p/n G2545A)

Hybridization oven rotator for Agilent Microarray Hybridization Chambers (Agilent p/n G2530-60029)

**Procedure:**

1. Add 500 μL of nuclease-free water to the vial containing lyophilized 10X Blocking Agent. Mix by gently vortexing.
2. Equilibrate water bath to 60°C.
3. For each microarray, add each of the components as indicated in the tables as below to a 1.5 mL nuclease-free microfuge tube:

| For 4*44K array | Amount |
| --- | --- |
| cyanine 3-labeled, linearly amplified cRNA | 1.65ug |
| 10X Blocking Agent | 11ul |
| Nuclease-free water | ×ul |
| 25X Fragmentation Buffer | 2.2ul |
| **Total volume** | **55ul** |

| For 8*60K array | Amount |
| --- | --- |
| cyanine 3-labeled, linearly amplified cRNA | 0.6ug |
| 10X Blocking Agent | 5ul |
| Nuclease-free water | ×ul |
| 25X Fragmentation Buffer | 1ul |
| **Total volume** | **25ul** |

1. Mix well but gently on a vortex mixer.
2. Incubate at 60°C for exactly 30 minutes to fragment RNA.
3. Add 2x GEx Hybridization Buffer HI-RPM to the array to stop the fragmentation reaction.

| **Volumes per hybridization** | | | |  |
| --- | --- | --- | --- | --- |
| Components | 4*44K | 8*60k |  | |
| cRNA from Fragmentation Mix | 55ul | 25ul |  | |
| 2* GEx Hybridization Buffer HI-RPM | 55ul | 25ul |  | |

1. Mix well by careful pipetting. Take care to avoid introducing bubbles. Do not mix on a vortex mixer; mixing on a vortex mixer introduces bubbles.
2. Spin for 1 minute at room temperature at 13,000 rpm in a microcentrifuge to drive the sample off the walls and lid and to aid in bubble reduction.
3. Place sample on ice and load onto the array as soon as possible.
4. Load a clean gasket slide into the Agilent SureHyb chamber base with the label facing up and aligned with the rectangular section of the chamber base. Ensure that the gasket slide is flush with the chamber base and is not ajar.
5. Slowly dispense the volume of hybridization sample onto the gasket well in a “drag and dispense” manner.

| **Volumes per hybridization** | | | |  |
| --- | --- | --- | --- | --- |
| Components | 4*44K | 8*60K |  | |
| Volume Prepared | 110ul | 50ul |  | |
| Hybridization Sample Volume | 100ul | 40ul |  | |

1. Slowly place an array “active side” down onto the SureHyb gasket slide, so that the “Agilent”-labeled barcode is facing down and the numeric barcode is facing up. Verify that the sandwich-pair is properly aligned.
2. Place the SureHyb chamber cover onto the sandwiched slides and slide the clamp assembly onto both pieces.
3. Hand-tighten the clamp onto the chamber.
4. Vertically rotate the assembled chamber to wet the gasket and assess the mobility of the bubbles.
5. Place assembled slide chamber in rotisserie in a hybridization oven set to 65°C. Set your hybridization rotator to rotate at 10 rpm.
6. Hybridize at 65°C for 17 hours.

# Step 5 Microarray Wash

**Kit and Instruments:**

Gene Expression Wash Buffer 1 (Agilent p/n 5188-5325)

Gene Expression Wash Buffer 2 (Agilent p/n 5188-5326)

Magnetic stir bar (Corning p/n 401435)

Magnetic stir plate (Corning p/n 6795-410)

Slide-staining dish, with slide rack (Thermo Shandon p/n 121)

**Procedure:**

1. Prewarm enough volume of Gene Expression Wash Buffer 2 to 37°C.
2. Add the slide rack and stir bar to the staining dish.
3. Transfer the staining dish with the slide rack and stir bar to a magnetic stir plate.
4. Fill the staining dish with 100% acetonitrile.
5. Turn on the magnetic stir plate and adjust the speed to a setting of 4 (medium speed).
6. Wash for 5 minutes.
7. Discard the acetonitrile as is appropriate for your site.
8. Repeat step 2 to step 7.
9. Air dry the staining dish in the vented fume hood.
10. Wash all dishes, racks, and stir bars with Milli-Q water.
11. Completely fill slide-staining dish #1 with Gene Expression Wash Buffer 1 at room temperature.
12. Place a slide rack into slide-staining dish #2. Add a magnetic stir bar. Fill slide-staining dish #2 with enough Gene Expression Wash Buffer 1 at room temperature to cover the slide rack. Place this dish on a magnetic stir plate.
13. Place the empty dish #3 on the stir plate and add a magnetic stir bar. Do not add the prewarmed (37°C) Gene Expression Wash Buffer 2 until the first wash step has begun.
14. Remove one hybridization chamber from incubator and record time. Record whether bubbles formed during hybridization and if all bubbles are rotating freely.
15. Prepare the hybridization chamber disassembly.
16. Place the hybridization chamber assembly on a flat surface and loosen the thumbscrew, turning counterclockwise.
17. Slide off the clamp assembly and remove the chamber cover.
18. With gloved fingers, remove the array-gasket sandwich from the chamber base by grabbing the slides from their ends. Keep the microarray slide numeric barcode facing up as you quickly transfer the sandwich to slide-staining dish #1.
19. Without letting go of the slides, submerge the array-gasket sandwich into slide-staining dish #1 containing Gene Expression Wash Buffer 1.
20. With the sandwich completely submerged in Gene Expression Wash Buffer 1, pry the sandwich open from the barcode end only:
21. Slip one of the blunt ends of the forceps between the slides.
22. Gently turn the forceps upwards or downwards to separate the slides.
23. Let the gasket slide drop to the bottom of the staining dish.
24. Remove the microarray slide and place into slide rack in the slide-staining dish #2 containing Gene Expression Wash Buffer 1 at room temperature.
25. When all slides in the group are placed into the slide rack in slide-staining dish #2, stir using setting 4 for 1 minute.
26. During this wash step, remove Gene Expression Wash Buffer 2 from the 37°C water bath and pour into the slide-staining dish #3.
27. Transfer slide rack to slide-staining dish #3 containing Gene Expression Wash Buffer 2 at elevated temperature. Stir using setting 4 for 1 minute.
28. Slowly remove the slide rack minimizing droplets on the slides. It should take 5 to 10 seconds to remove the slide rack.
29. Scan slides immediately to minimize the impact of environmental oxidants on signal intensities.

# Step 6 Scanning

**Instrument:**

Agilent Microarray Scanner (Agilent p/n G2505C)

**Procedure:**

1. Assemble the slides into an slide holder.
2. Place assembled slide holders into scanner carousel.
3. Verify scan settings for one-color scans.

|  | Parameters |
| --- | --- |
| Scan region | Scan Area (61 x 21.6 mm) |
| Scan resolution (μm) | 5 |
| 5μm scanning mode | Single Pass |
| eXtended Dynamic range | (selected) |
| Dye channel | Green |
| Green PMT | XDR Hi 100%  XDR Lo 10% |

1. Click **Scan Slot m-n** on the Scan Control main window where the letter **m** represents the Start slot where the first slide is located and the letter **n** represents the End slot where the last slide is located.

# Step 7 Extract data using Agilent Feature Extraction Software

**Software:**

Agilent Feature Extraction

**Procedure:**

1. Open the Agilent Feature Extraction (FE) software.
2. Add the images (.tif) to be extracted to the FE Project.
3. Set FE Project Properties.
4. Check the Extraction Set Configuration.
5. Save the FE Project (.fep) by selecting **File > Save As** and browse for desired location.
6. Select **Project > Start Extracting** and export data to txt.
