# Supplementary figures and images for: Combined identification of lncRNA NONHSAG004550 and NONHSAT125420 as a potential diagnostic biomarker of perinatal depression
Source: J Clin Lab Anal. 2021 Jul 15;35(8):e23890. doi: 10.1002/jcla.23890 (PMC8373316; doi:10.1002/jcla.23890)

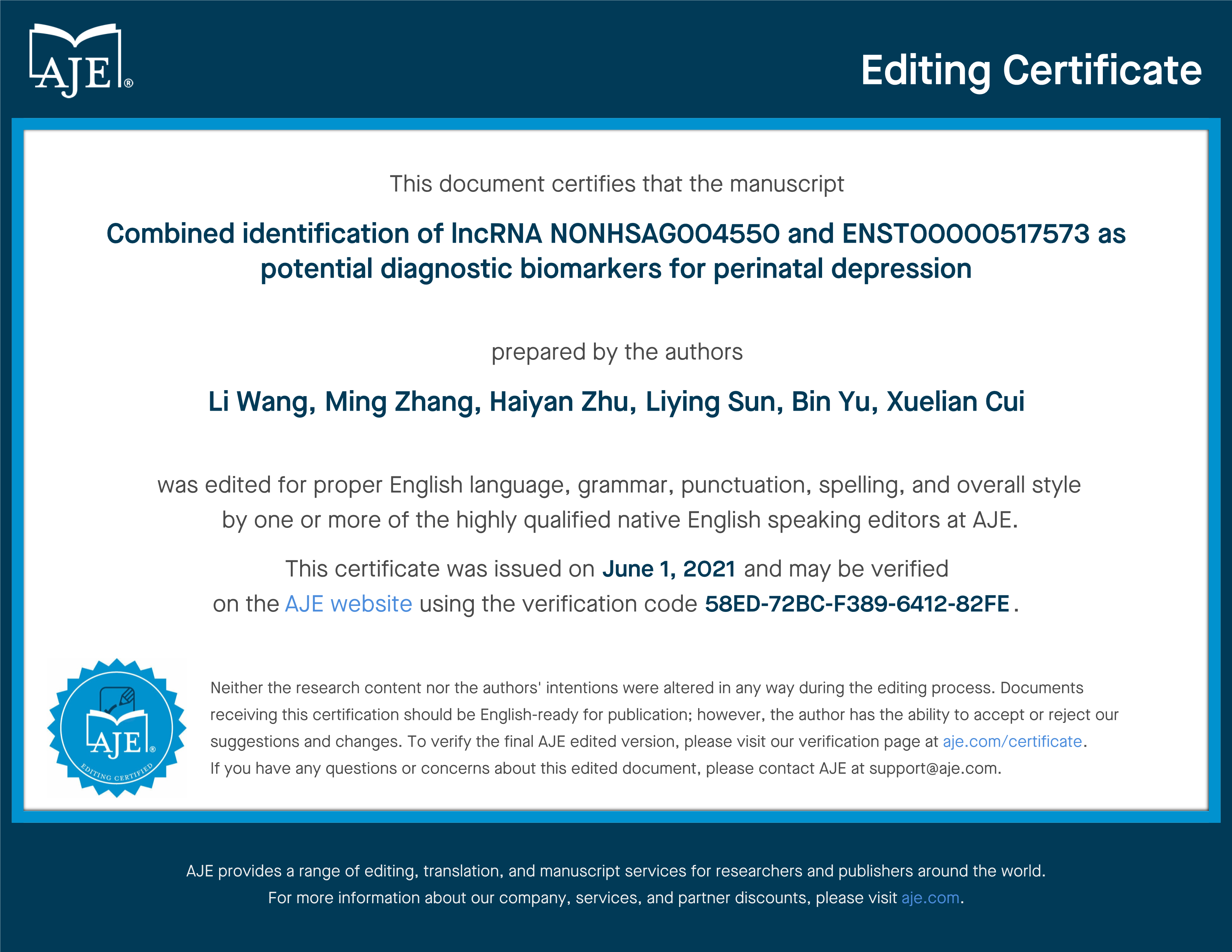

Supplement: Supplementary file 2 — Supplementary Material [file JCLA-35-e23890-s001.tif]
